# Performance Evaluation on Rotary Preparation of Root Canal by Beginner Operators

**DOI:** 10.25122/jml-2020-0136

**Published:** 2020

**Authors:** Mihaela Chirila, Ioana Suciu, Bogdan Dimitriu, Nicoleta Maru, Ecaterina Ionescu, Georgiana Florentina Croitoru, Oana Amza

**Affiliations:** 1. Department of Endodontics, Faculty of Dental Medicine, “Carol Davila” University of Medicine and Pharmacy, Bucharest, Romania; 2. Department of Clinical Anatomy and Topography, “Carol Davila” University of Medicine and Pharmacy, Bucharest, Romania; 3. Department of Orthodontics and Dento-Facial Orthopedics, Faculty of Dental Medicine,“Carol Davila” University of Medicine and Pharmacy, Bucharest, Romania

**Keywords:** Pre-clinical, ProTaper Universal, endo training blocks

## Abstract

This study aims to analyze the performance of rotary root canal preparation, conducted by beginner operators (students). After acquiring the necessary skills involved in applying endodontic preparation techniques on extracted teeth, all students from a cohort learned to use ProTaper Universal files (Dentsply Maillefer). The preclinical training had several stages. Experience 1: instrumentation on extracted single-root teeth; Experience 2: instrumentation on EndoTraining blocks (Dentsply Maillefer); Experience 3: instrumentation on EndoTraining blocks (Dia Dent Group International); Experience 4: Instrumentation on extracted multiradicular teeth. Preparation was done according to the manufacturer’s instructions, the initial file is Sx, followed by S1, S2, F1, F2 and the last file is F3. A batch of 50 teeth (E1, E2, E3, E4) was randomly selected to evaluate the onset of the rotary preparation of young operators. Two independent evaluators analyzed the array radiologically by stereomicroscope evaluation (E1, E4) and photo-analysis of the resulting Endo Training blocks (E2, E3). The success rate was 80% for E1, 72% for E2, 64% for E3 and 76% for E4 (p<0.05). The following were considered a failure: ledge formation and apical transportation in 10.66%, over instrumentation in 6.66%, zipping in 3.33%, and instrument fracture in 4% of cases.

Endodontic instrumentation techniques require training to acquire the necessary skills. Rotary root canal instrumentation techniques used almost exclusively in modern endodontics require adequate preclinical training.

## Introduction

Endodontic treatment involves cleaning and shaping the root canals. By preparing the root canals, the organic and inorganic tissues present at this level are removed, and by disinfection, the number of microorganisms is reduced, and the endotoxins inside the dentinal wall are neutralized and the canal acquires an adequate shape in order to achieve canal filling [1].

Particularly important in endodontic treatment is the observance and preservation of the shape and initial direction of the root canal throughout the preparation [2].

Stainless steel tools have been used to instrument root canals in endodontics. In order to improve the efficiency of instrumentation in the preparation of root canals, endodontic techniques and instruments are perfected [3]. The new technologies aim to reduce possible unwanted clinical errors such as transportation, ledges, strip perforations, canal straightening or fracture of the instrument [4].

In the last decade, the development and improvement of rotary instrumentation systems have determined the simplification of root canal preparation procedures, reducing risks and working time [2].

The instrumentation of the root canals can be done with manually-operated or machine-assisted instruments. In the last decades, rotary instrumentation became more manageable and faster, thus growing in popularity. This type of instrumentation requires more flexible nickel-titanium (NiTi) instruments. By comparison, those of the first generation had negative rake angles in an attempt to avoid taper lock and prevent the transportation of the canal system and were used in a crown-down sequence [5].

Compared to stainless steel needles, NiTi instruments have the specific characteristics of the alloy: lower modulus of elasticity, shape memory and super-elastic behavior. Consequently, in the preparation of narrow and curved root canals, they are more conservative and efficient [2].

The ProTaper Universal system (Dentsply Maillefer, Ballaigues, Switzerland) is currently the rotary instrumentation system of choice for most endodontists. The ProTaper Universal (PTU) is a NiTi rotary instrumentation system with a progressively tapered design, aiming to improve cutting efficiency, flexibility, and safety. PTU instruments relate to their convex triangular cross-section, which enhances the cutting action while keeping the friction between the file and dentin to a minimum. ProTaper Universal instruments have changing spiral parameters (angle and pitch) over their cutting edges, thus reducing the chance of an instrument scratching the canal surface accidentally. However, it was found that this type of alloy was not solely responsible for the recorded improvements in canal preparation. A plethora of factors influenced the instrument properties and their performance [6].

Ex vivo studies on extracted teeth generally performed by a single experienced dentist have shown that rotary instrumentation is safe, preparation is rapid, and generally respects the initial anatomy of the root canal [5]. The operator’s experience on root canal instrumentation has scarcely been investigated in specialized studies. However, some studies have shown that rotary systems can be used properly by novice operators [7].

## Material and Methods

All students in a series went through the basics of endodontic instrumentation techniques by using the K-file and PTU manually. After mastering the necessary skills, which involved applying manual endodontic preparation techniques on extracted teeth and endo-blocks, the students understood how to use the ProTaper system. The rotary technique of endodontic preparation using PTU needles has been explained and demonstrated. Each student handled for the first time extracted teeth and endo-blocks in succession:

• Experience 1 (E1). Instrumentation on extracted single-root teeth;
• Experience 2 (E2). Instrumentation on Endo Training blocks (Dentsply Maillefer);
• Experience 3 (E3). Instrumentation on Endo Training blocks (Dia Dent Group International);
• Experience 4 (E4). Instrumentation on extracted multiradicular teeth.

For experience 1 and 4, all extracted teeth used were previously disinfected with sodium hypochlorite. The extracted teeth to be used for root canal preparation were approved by supervisors. The selected teeth did not present an accentuated curvature, a narrowing shape, or gross dentin depositions. 

First, preoperative radiographs were made. After the cavity access and canal permeabilization, second radiographs were made to establish the preliminary odontometry study. 

For experience 2, we used the single curvature Endo Training blocks (Dentsply Maillefer); 16 mm canal length with a 0.02 taper, International Standards Organization (ISO) size 15, and a 45° curvature using Schneider’s method [7].

For experience 3, we used the single curvature Endo Training blocks with two accessory channels (Dia Dent Group International); 16 mm canal length with a 0.04 taper, ISO size 15, and a 45° curvature using Schneider’s method [7].

The first steps in using NiTi files are determining the working length and preparing a glide path with a size 10 SS K-file. 

The instrumentation of the root canals was done with a PTU system using the sequence Sx, S1, S2, F1, F2, F3, using the crown-down technique. The endodontic motor X-Smart Plus, with a 16:1 reduction handpiece, was used, ensuring a continuous clockwise rotation of the files. The torque settings and rotation speed followed the manufacturer’s guidelines.

The endodontic engine was set at 300 rotations per minute (RPM) with 2 Ncm torque and an Sx file was used in the coronal part of the canal. First, S1 and S2 were used with brushing motions in the two-thirds of the canal and then at the entire working length (WL). F1 (20.07), F2 (25.08), and F3 (30.09) were used with in-and-out pecking motions until the full WL was reached. 2 mL of distilled water was used as an irrigator at each change of instrument.

A batch of 50 teeth was randomly selected and was evaluated in a double-blind manner (by two independent evaluators). We analyzed the apical surface of each tooth using a stereomicroscope with a 10X magnification (Olympus camera). Finally, radiographs were used for the qualitative assessment of root canal preparation.

Blocks were photographed with a Nikon D7000 camera in a fixed position before and after the root canal instrumentation.

## Results

**Figure 1:**
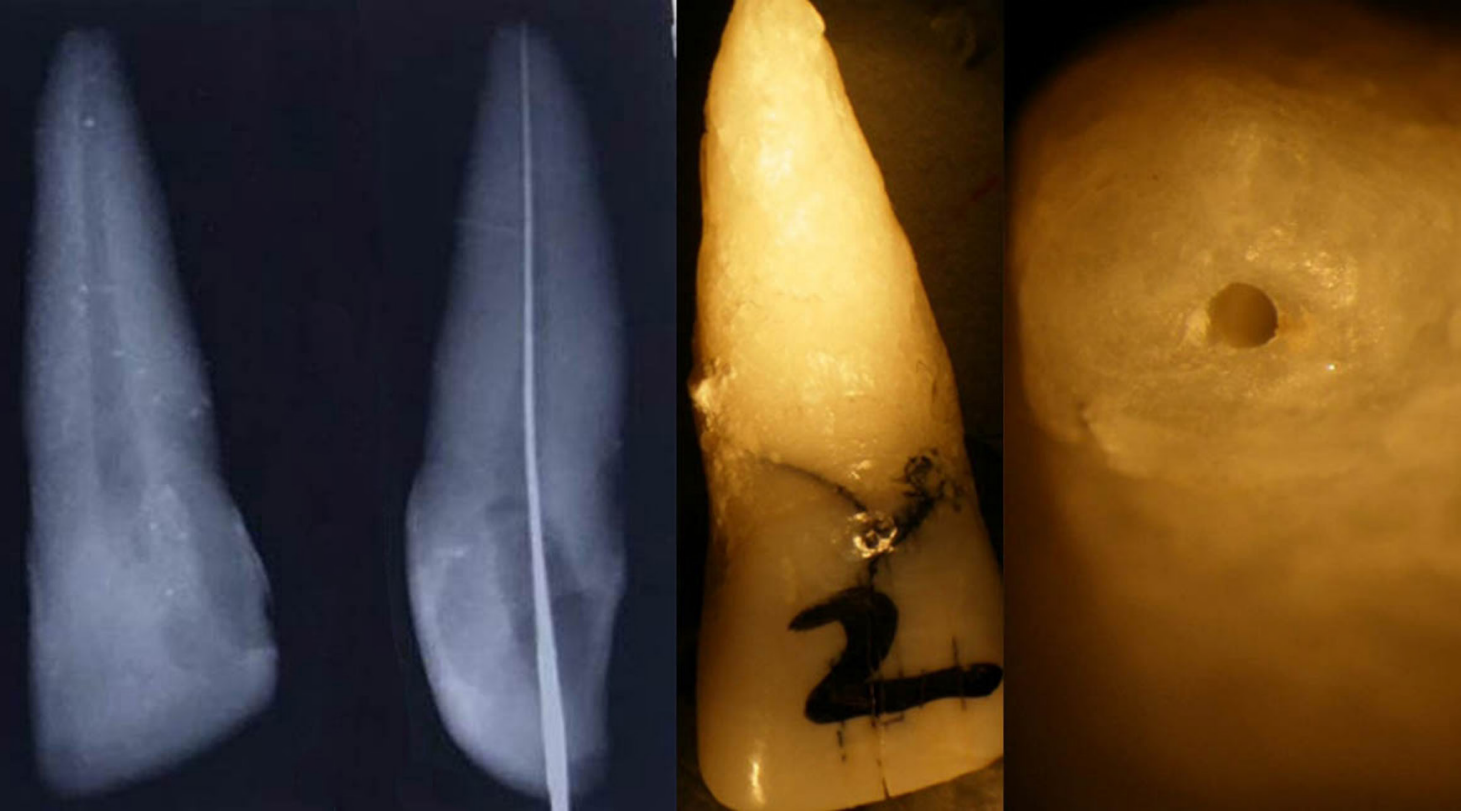
Experience 1. Instrumentation of 2.1 with rotary PTU. a,b,c pre-treatment, d- post-treatment apical over-preparation. Image taken using a stereomicroscope.

**Figure 2:**
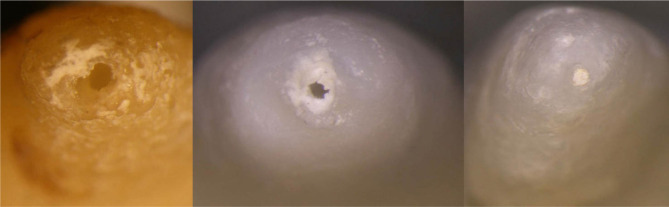
Experience 1. a,b – apical transposition, c- unharmed apex. Image taken using a stereomicroscope.

**Figure 3:**
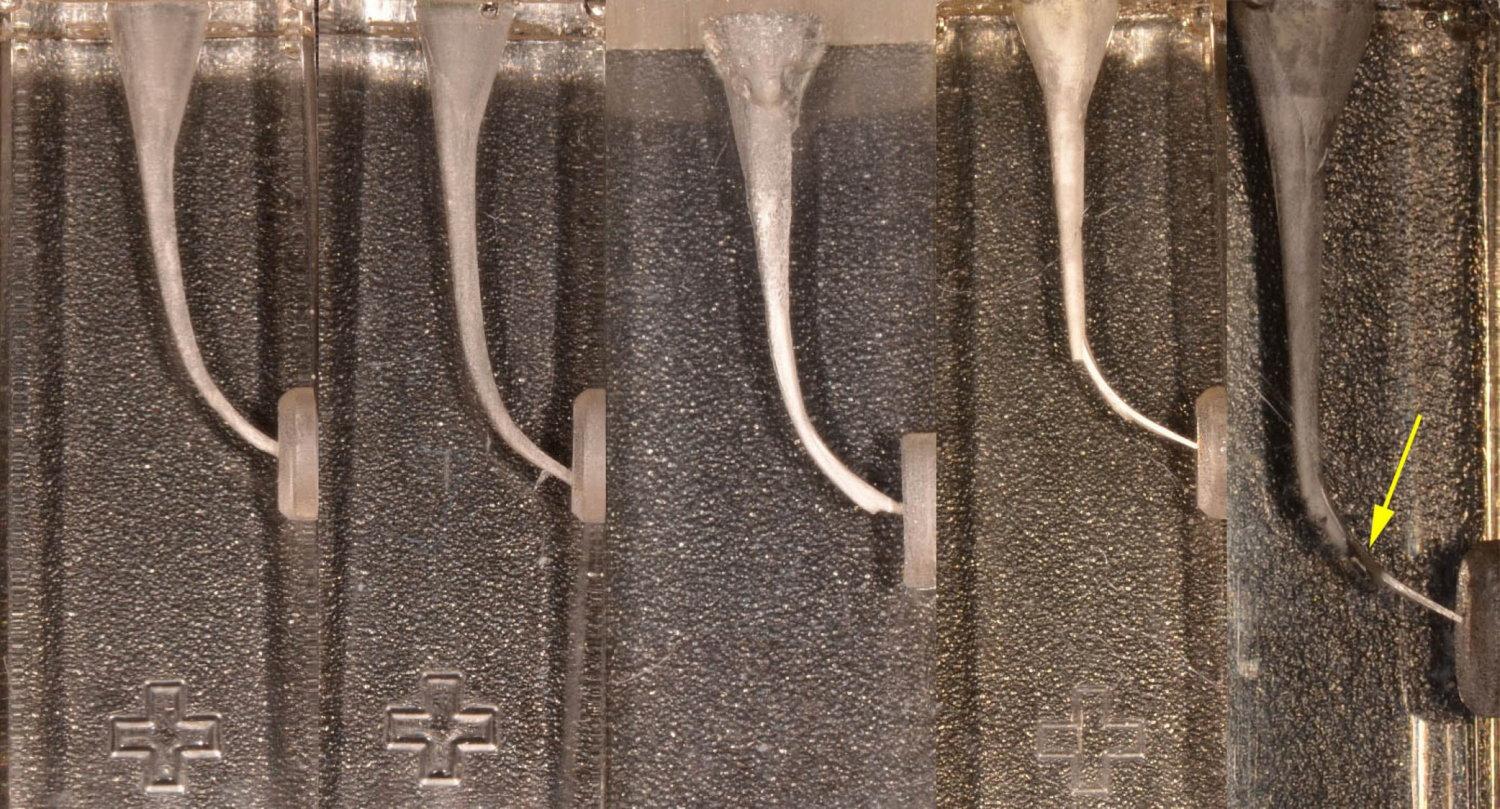
Experience 2. Instrumentation on Endo Training blocks (Dentsply Maillefer), a- correct preparation, b- apical transportation, c- ledge and zipping, d- ledge, e- instrument separated.

**Figure 4:**
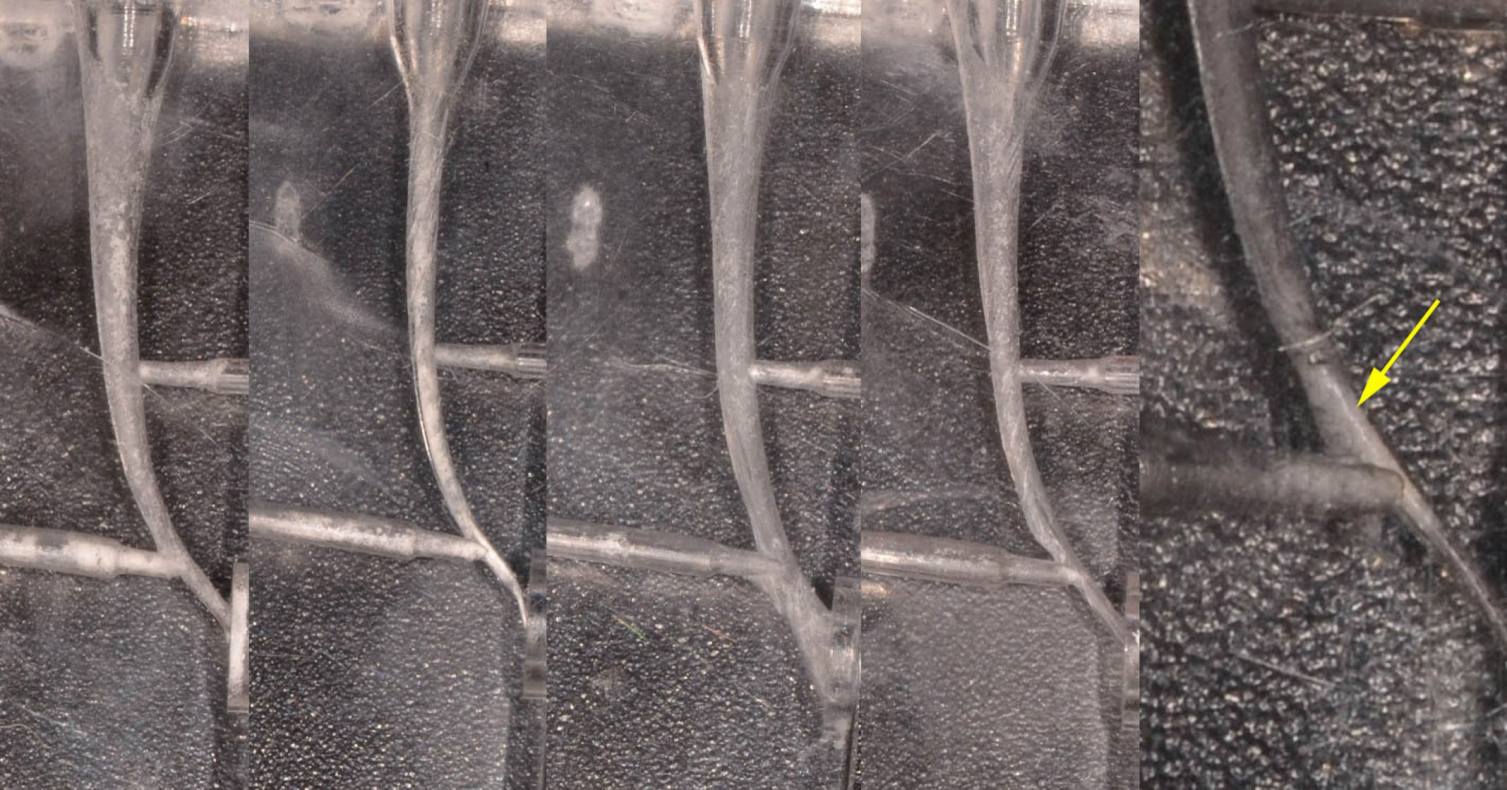
Experience 3. Instrumentation on Endo Training blocks (Dia Dent Group International), a- correct preparation, b-ledge, c – apical zipping, d - ledge, e- instrument separated.

**Figure 5:**
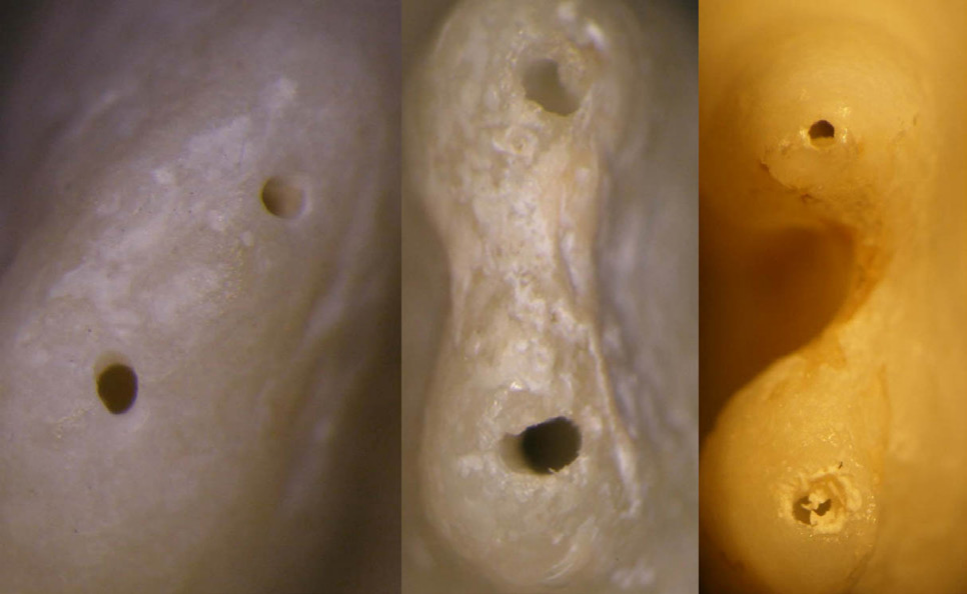
Experience 4. Instrumentation on bicuspid extracted tooth. a,c - post-treatment apical over-prepartion, b- apical transportation. Image taken using a stereomicroscope.

**Figure 6:**
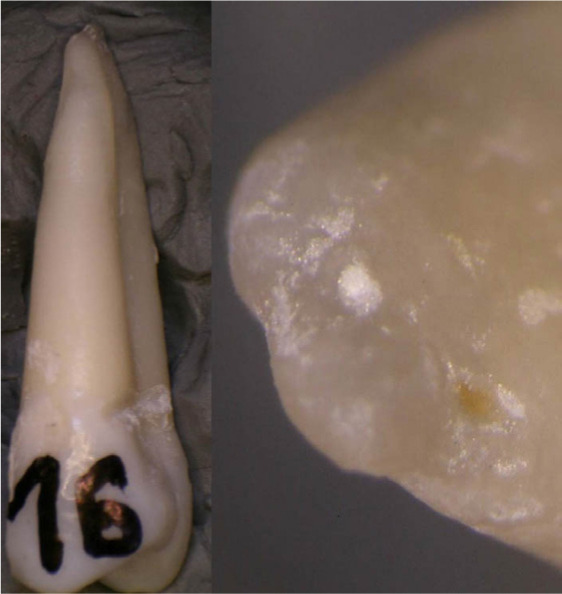
Experience 4. Instrumentation on bicuspid extracted tooth. Image taken using a stereomicroscope. Apex correctly prepared.

The images were assessed for ledges, apical zipping, separated instruments, canal transportation, and over-preparation, as described by Cohen and Hargreaves [8]. Success is defined as a block being prepared without error and as close to the ideal preparation as possible. If any of the above errors were identified, that specific sample would be considered as a failure. Once each of the two observers had completed the assessments, any disagreement was discussed jointly, and a consensus was reached. A selection of images from this study showing the ideal preparation, and each procedural error are shown in Figures 1-6.

Figure 1. Experience 1. Instrumentation of 2.1 with rotary PTU. a,b,c pre-treatment, d- post-treatment apical over-preparation. Image taken using a stereomicroscope.

Data gathered was converted into percentages and subsequently into success/failure odds. The Z-test was performed on the resulting probability ratios. Since the test was performed on a relatively small dataset formed of ratios, the confidence interval was logarithmic (natural log). The significance threshold was set at 95% (alpha = 0.95).

The p-values were computed assuming a uniform distribution across the populations, E1: p<0.00001; E2: p=0.00015; E3: p=0.003; E4: p<0.00001.

## Discussion

**Figure 7:**
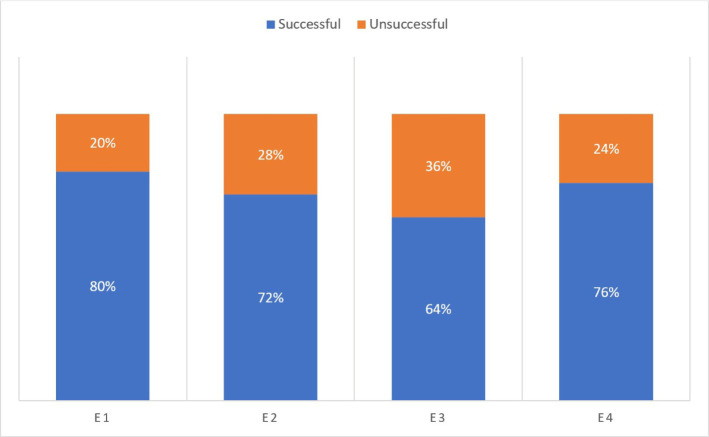
Succes percentage of experience using rotary ProTaper Universal in root canal instrumentation by beginner operators.

Our study evaluated the performance of the initial trial of the universal ProTaper rotary system by 50 randomly selected students. Conceived as realistic as possible, with a gradual increase in difficulty, it included extracted teeth and Endo Training blocks favoring the gradual progress and the most thorough acquisition abilities of the preparation technique (Figure 7).

In addition to hand files, instrumentation with rotary files is also considered necessary. For students, one problem was the difficulty of finding suitable teeth. The morphological variability and the difficulty of treating extracted teeth often determined the occurrence of errors and accidents, altering the students’ individual performance.

A problem which may have great implications is that the teeth used in the didactic process can be a source of infectious materials, both on the root surface and inside the radicular canals. Disinfection of the extracted teeth must be done, and that can potentially be a time-consuming process [4]. In certain situations, it is better to choose artificial teeth for novice practitioners in order to avoid working with hazardous materials [9]. Resin-based models are often used in academic environments, as they present standard canal specifications. [4]

The instrumentation of PTU is taught today in most faculties in Europe and represents the technique against which any new technique is compared.

The European Society of Endodontology (ESE) Education and Scholarship Committee encourages innovation and sharing technological advancements through current learning programs [10]. NiTi rotary tools can be used in conjunction with ISO standardized hand tools. The effects of the metal alloy, tip design, cutting face, cross-section, chip space, and taper on an instrument’s performance must be understood by students. Learning these principles is the foundation that allows students to later set benchmarks in choosing other rotary instrumentation systems [5].

According to some authors, the results obtained by rotating endodontic instrumentation and investigated in several studies were similar to those obtained by manual instrumentation [5].

Analysis of the preparation efficiency in the present study shows an increase in apical transport errors correlated with canal curvature while over-instrumentation decreased for the same reason.

**Figure 8:**
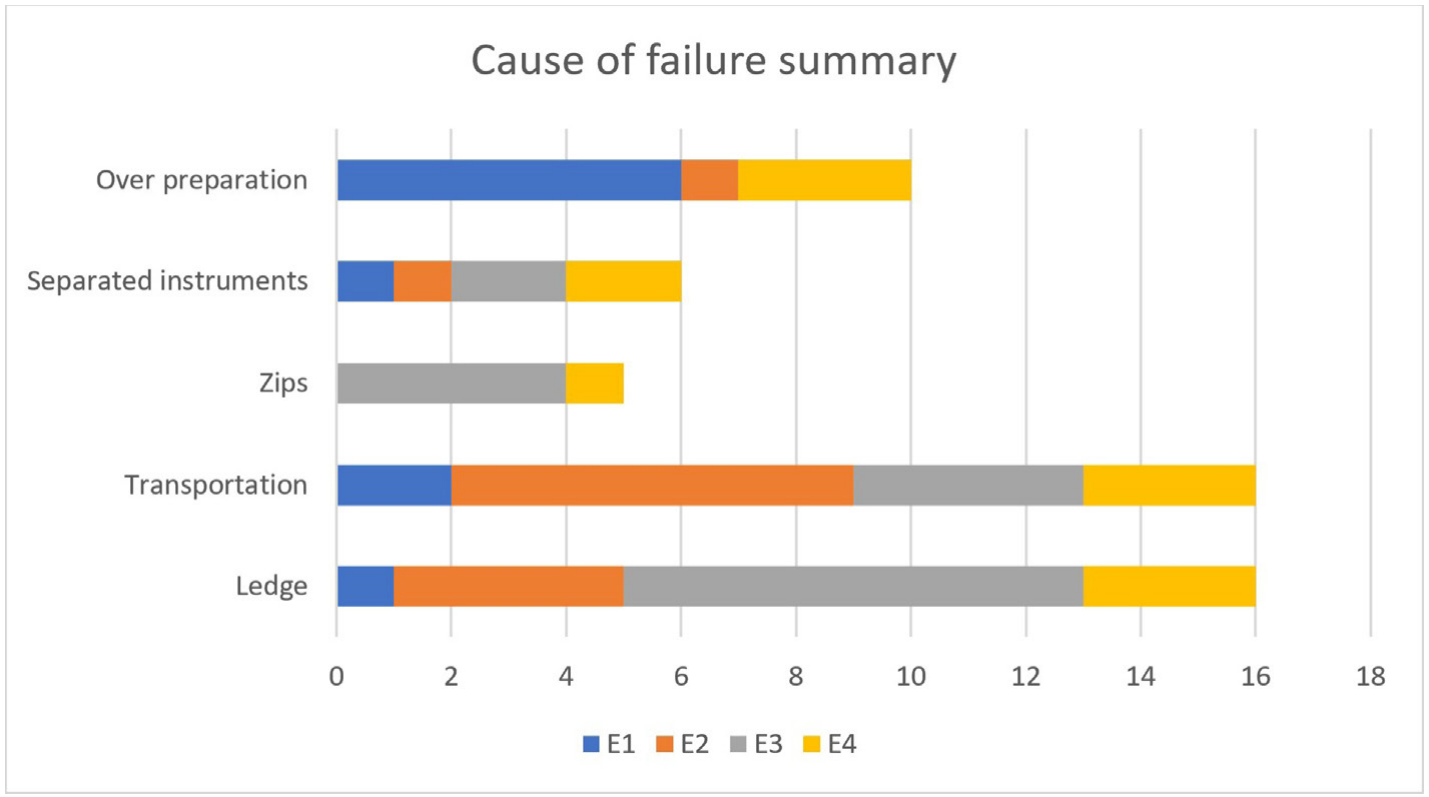
Cause of failure on rotary preparation of root canal by beginner operators.

Studies have reported that inexperienced undergraduate students produced less apical transportation with F1 and F2 PTU files in arched canals [11]. In our study, the apical transportation was 10.66% and over-instrumentation was noted in 6.66% of cases (Figure 8). Low fracture rates (4%) were observed in the E3 and E4 experiences. The Endo Training blocks (E3) (Dia Dent Group International) had a higher degree of difficulty and proved to be more fragile during instrumentation. Another cause of fracture of rotating Ni-Ti instruments can be cyclic fatigue [1,3,5]. Recent studies have concluded that the rotary systems examined can be used safely by students and novice practitioners [12]. In our study, deviations from the original canal curvature in the apical third were observed in 10.66% of the canals prepared with PTU. Compared to other studies, this ledge was recorded in 19% of cases [4]. 

The resin block is also transparent, which allowed assessment using trans-illumination. Due to the differences between resin and dentin, the results obtained in studies performed on simulated channels must be extrapolated clinically with great care [13].

## Conclusion

The use of Ni-Ti rotary instruments provides an acceptable level of canal shaping for beginner operators. The preclinical trainer uses rotary instruments and consequently improves the clinical experience of students.

## Conflict of Interest

The authors declare that there is no conflict of interest.
